# A novel carcinogenic mouse model by site‐directed insertion of tandem human *HRAS* large DNA fragment into 15E1 site

**DOI:** 10.1002/ame2.70086

**Published:** 2025-11-12

**Authors:** Susu Liu, Yanwei Yang, Guitao Huo, Hao Yang Zhao, Chen Ling, YuYa Wang, Shijie Zhai, Xiaowei Sun, Wenda Gu, Yuan Cao, Wei Gong, Sanlong Wang, Changfa Fan

**Affiliations:** ^1^ Division of Animal Model Research, Institute for Laboratory Animal Resources National Institutes for Food and Drug Control (NIFDC) Beijing China; ^2^ National Rodent Laboratory Animal Resource Center Beijing China; ^3^ National Centre for Safety Evaluation of Drugs, Institute for Food and Drug Safety Evaluation National Institutes for Food and Drug Control (NIFDC) Beijing China

**Keywords:** human *HRAS* gene, ICH S1B guideline, KI.C57‐ras carcinogenic mouse model, large DNA fragment editing, non‐clinical carcinogenicity evaluation

## Abstract

**Background:**

The precise insertion of large DNA fragments (>3–5 kb) remains one of the key obstacles in establishment of genetically modified murine models.

**Methods:**

A 21 kb large DNA fragment containing three tandemly linked copies of the human *HRAS* gene was inserted into the genome of C57BL/6J mouse, generating a mouse model designated as KI.C57‐ras (or named NF‐hHRAS). Whole‐genome sequencing and Sanger sequencing were utilized to it confirm precise insertion and copy number. The stability of transgene expression among different generations was verified from multiple aspects using by digital PCR, western blot and DNA sequencing. To assess tumor susceptibility in the mouse model, N‐Nitroso‐N‐methylurea (MNU) was administered at a dosage of 75 mg/kg. Histopathological examinations were conducted using hematoxylin and eosin (H&E) staining.

**Results:**

The *HRAS* DNA fragment was inserted into mouse chromosome 15E1 site, locating between 80 623 202 bp and 80 625 020 bp. NF‐hHRAS mice exhibited stable inheritance and displayed consistent phenotypes across individuals. Moreover, this mouse model exhibited a high susceptibility to carcinogens. Upon administration of MNU the earliest mortality onset was earlier than that of wild‐type littermates (day 65 vs. day 78 for male and day 56 vs. day 84 for female). Notably, 100% of the NF‐hHRAS transgenic mice developed tumors, with approximately 84% of male NF‐hHRAS mice exhibiting specific tumor types, such as squamous cell carcinoma or squamous cell papilloma, which was consistent with the previously reported carcinogenic rasH2 mouse model. The types of tumors and the target organs exhibited diversity in NF‐hHRAS mice, while the spontaneous tumor incidence remained low (1/50).

**Conclusions:**

The NF‐hHRAS mice demonstrated excellent genetic stability, a reproducible phenotype, and high susceptibility to carcinogens, indicating their potential utility in non‐clinical safety evaluations of drugs as per the S1B guidelines issued by the ICH (The International Council for Harmonization of Technical Requirements for Pharmaceuticals for Human Use).

## Graphical abstract

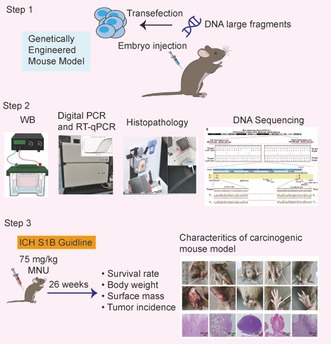

1. The NF‐hHRAS mouse model was generated through Chr15 E1 locus‐targeted integration of a 21 kb multicopy HRAS gene cluster.
2. The NF‐hHRAS model demostrated tissue‐specific expression of human HRAS across multiple organs and low spontaneous tumorigenesis.
3. Preliminary validation supports the NF‐hHRAS model's utility in nonclinical oncogenicity risk evaluation under ICH S1B guidelines.

## 1 INTRODUCTION

Genome editing technology has emerged as one of the most transformative and disruptive innovations in various fields of biotechnology. Following decades of iterative advancements, genome editing tools have evolved to enable rapid and precise manipulation of DNA sequences, including gene knockout, site‐directed mutagenesis, and the insertion of small DNA fragments. The advent of transgenic technology in 1980 marked the onset of this revolutionary era, followed seven years later by the development of embryonic stem (ES) cell‐based gene targeting.^1^, ^2^ Subsequently, programmable nuclease‐dependent systems, including zinc finger nucleases (ZFNs) and transcription activator‐like effector nucleases (TALENs), were developed in the 1990s,^3^ significantly improving the efficiency homology‐directed repair (HDR). In 2013, CRISPR‐Cas9 was recognized for its exceptional efficiency, simplicity, and programmability, becoming the most predominant genome‐editing platform.^4^, ^5^ Furthermore, several innovative systems have been developed to address specific genome‐editing challenges. For instance, the homology‐independent targeted integration (HITI) system enables large‐fragment integration via non‐homologous end joining.^6^ Prime Editing (2019) facilitates precise small‐fragment insertions and deletions.^7^, ^8^ The programmable addition via site‐specific targeting elements (PASTE) integrated integrase system achieves kilobase‐scale site‐specific integration in mammalian cells.^9^ Additionally, CAST transposase‐based platforms enable DNA cleavage‐independent integration, although their efficiency remains limited in eukaryotic systems.^10^, ^11^

The precise editing of large genome DNA fragments (exceeding 3–5 kb) in mammals, such as rodent models, is urgently required but currently limited by low efficiency.^12^ Previous studies have shown innovative research in this area, including homology‐mediated end joining (HMEJ) for high‐efficiency integration via optimized donor design,^13^, ^14^ transcription‐coupled donor delivery (TED),^10^, ^15^ single/double‐stranded template‐based editing (LOCK),^16^ prime editing‐driven writing (PAINT),^17^ and the PrimeRoot system that integrates prime editing with Cre recombinase.^18^ In comparison to the novel systems, ES cell‐based targeting, despite being technically demanding and resource‐intensive, offers unique advantages for large‐fragment integration.

The human *RAS* oncogene family, comprising *HRAS*, *KRAS*, and *NRAS*, encodes RAS proteins with GTPase activity.^19^ The active RAS protein interacts with effector molecules and regulates critical cellular processes via the PI3K/AKT and RAF/MEK/ERK signaling pathways, including glycolysis, the tricarboxylic acid (TCA) cycle, and lipid metabolism.^20^ Previous research has demonstrated that the traditional transgenic technology to develop a genetically modified carcinogenic mouse model, rasH2, by random integrating the human *HRAS* oncogene (*c‐Ha‐ras*) into the genome of C57BL/6J mice. This model has been widely utilized globally for the non‐clinical assessment of potential carcinogenicity in drug candidates.

To validate the reliability of ES cell‐based large‐fragment integration and to develop a novel site‐directed insertion carcinogenic mouse model for non‐clinical drug evaluations, a large DNA fragment containing three tandem copies of the human *c‐Ha‐ras* gene was precisely integrated into the Chr15‐E1 locus. Comprehensive characterizations, including sensitivity to carcinogen, tumor spectrum and spontaneous tumor incidence, were assessed.

## 2 MATERIALS AND METHODS

### 2.1 Karyotype analysis and Southern blot of ES cell lines

For karyotyping analysis, mouse embryonic stem (ES) cells were seeded into 6‐well plates at a density of 1 × 10^6^ cells per well. One day post‐seeding, the culture was treated with 0.5 mg/mL colcemid (Sigma) and incubated for 50 min at 37℃ in a water bath. Subsequently, the mouses ES cells were dissociated using trypsin, fixed with a methanol‐glacial acetic acid (3:1) solution, and spread onto glass‐slides. Chromosome G‐binding was analyzed for karyotypes by microscopy.

Genomic DNA was extracted from ES cells using the phenol‐chloride extraction method. 25 μg of genomic DNA was digested with *BamHI* (NEB) at 37℃ overnight. Following electrophoresis and membrane transfer, the expected band was detected with ^32^P‐labeled probes (Promega) at 67℃ overnight.

### 2.2 Generation of the NF‐hHRAS mouse model

We synthesized the full‐length 7.0 kb genomic DNA (gDNA) of the human wild‐type *c‐Ha‐ras* (Genbank No. 3265). Subsequently, three tandem copies of *c‐Ha‐ras* were linked together into a 21 kb large DNA fragment via BAC in vivo recombination technology. To craft the targeting vector, we incorporated three copies of gDNA encoding human HRAS protein into the pDTA vector, which also contained the homologous arm sequences of the 15E1 recombination site along with a neomycin selection marker. The targeting vector was then electroporated into C57BL/N ES cells. Following this, G418‐resistant colonies were selected and propagated for further analysis. PCR analysis with primer set 1 (Table S1) was conducted to identify ES cells clones that undergone homologous recombination. Male chimeric mice were then crossed with C57BL/6J female mice to generate offspring harboring three tandem copies of the human *HRAS* genes, thereby establishing the C57BL/6J‐Chr15E1tm1 ^(*hHRAS‐hHRAS‐hHRAS*)^/Nifdc model (generic name: KI.C57‐ras or NF‐hHRAS mice). This model is characterized by the human *HRAS* gene being regulated by its endogenous enhancer and promoter.

### 2.3 Reverse transcription‐quantitative real‐time polymerase chain reaction (RT‐qPCR)

To evaluate gene expression levels by RT‐qPCR, total RNA was extracted from NF‐hHRAS mice tissues using the TRizol reagent (TAKARA, Japan) following the manufacturer instructions. Then, the RNA was reverse‐transcribed into cDNA using the RT‐PCR kit (TAKARA, Japan). The synthesized cDNA was subjected to real‐time PCR analysis (Light Cycler 480 Real‐Time PCR system) using primer set 3. *Gapdh* served as the endogenous control. Each reaction was performed in triplicate. Furthermore, total RNA was extracted from the heart, liver, spleen, lung, kidney, and stomach tissues of F_2_ and F_8_ NF‐hHRAS mice for relative quantification by RT‐qPCR.

### 2.4 Protein extraction and Western blot analysis

To extract total proteins, tissues were lysed in RIPA buffer containing a protease inhibitor and a phosphatase inhibitor. The BCA Protein Assay Kit (Sangon, China) was used to detect the protein concentration in tissues of NF‐hHRAS mice. Protein solutions were boiled in 5 × loading buffer at 98℃ for 10 min and then resolved by SDS‐PAGE. All the primary antibodies used in the experiment were: anti HRAS (1:1000, Proteintech); anti‐GAPDH (1:10 000; Sino biological). Primary antibodies were detected by goat anti‐rabbit IgG (1:5000).

### 2.5 Digital PCR

Lung tissue cDNA from F_2_ and F_8_ generation mice was quantified and diluted to a concentration of 10 ng/μL. Subsequently, 1 μL of the diluted cDNA was added to the PCR reaction mixture. After vortex, the 25 μL digital PCR mix was directly added to each well of the Sapphire Chip and then was put in the Naica Geode (Stilla Technologies) for droplet generation and amplification. The amplification program is 37℃ 10 min, 95℃ 10 min, 50 cycles of 95℃ 30 s and 62℃ 1 min. Naica™ Prism was used for data acquisition. And data analysis was performed on Crystal Miner software.

### 2.6 DNA sequencing

Three mice from each of the F_2_ and F_8_ generations were selected for genomic DNA extraction. The target fragments were amplified by PCR. After purification, the PCR products were sequenced using the Sanger sequencing method. The sequencing chromatograms were analyzed using Chromas software. Then, the sequence of the target fragment was aligned with the sequence of the targeting vector using BLAST to confirm the precise insertion site.

### 2.7 Whole‐genome sequencing (WGS)

To confirmed the integration site of the *HRAS* gene, the 5′‐end left and 3′‐end right homologous arms were identified by whole‐genome sequencing. DNA extraction and optional length sorting were performed on the sample to obtain DNA suitable for long‐read nanopore sequencing. The quality of the extracted DNA was assessed by measuring the A_260_/A_280_ ratio. The deoxyadenosine (dA) tails were added on the ends of DNA fragments by end repair reagents. Low‐speed sequencing mode was used to generate the whole genome sequencing data on the CycloneSEQ platform. We used minimap2 version 2.24‐r1164‐dirty to align reads to a reference genome.

### 2.8 Carcinogenicity test of the NF‐CB6F1 (hHRAS ^+/−^) mice

F_1_ hybrid mice of NF‐hHRAS (with C57BL/6J background) and BALB/c, namely NF‐CB6F1 (hHRAS^+/−^) mice, are usually used for carcinogenicity experiments. Male NF‐hHRAS^+/−^ mice were mated with female BALB/c mice to produce offspring. By genotype identification, hHRAS transgene positive and negative mice were separated, which were named NF‐CB6F1 (hHRAS^+/−^) and NF‐CB6F1 (hHRAS^−/−^) mice, respectively.

Carcinogenicity test was designed in accordance with the S1B guidelines issued by the ICH (The International Council for Harmonization of Technical Requirements for Pharmaceuticals for Human Use) and the testing items were executed based on the evaluation content of drug carcinogenicity. 6–8 weeks old mice with 25 mice of each gender were selected (Table 1). Each mouse was administered a single dose of positive carcinogen N‐Nitroso‐N‐methylurea (MNU) at 75 mg/kg body weight or solvent, and then following a continued observation last for 26 weeks. The preparation and administration method of MNU solution was referred to reference.^21^ General clinical signs were observed daily, body weight was measured weekly, and survival rates were recorded. Tumor incidence and progression were assessed by documenting the time of tumor onset, anatomical location, size and progression status.

**TABLE 1:** Experimental design and animal grouping.

Group number	Species	Number (F/M)	Route of administration	Positive carcinogen	Dose (mg/kg)
Group 1	CB6F1 (hHRAS^+/−^)	25/25	Intraperitoneal	MNU	75
Group 2	CB6F1 (hHRAS^−/−^)	25/25	Intraperitoneal	MNU	75

### 2.9 Histopathological examination

Moribund animals underwent emergency necropsy and pathological examination, dead animals were subjected to immediate postmortem necropsy, and surviving animals underwent scheduled necropsy at the end of the experiment. Tumor tissues and abnormal organs were fixed in 10% neutral buffered formalin. After fixation, tissues were trimmed, dehydrated through a graded ethanol series, embedded in paraffin, and sectioned at 3 μm thickness and stained with hematoxylin and eosin (H&E) for light microscopic examination. The tissue samples included brain, heart, liver, spleen, kidneys, lung, thymus, esophagus, stomach, duodenum, jejunum, ileum, cecum, colon, lymph nodes (mesenteric lymph nodes, inguinal lymph nodes, and submandibular lymph nodes), eyes, tongue, spinal cord, reproductive organs, skin and tissues with any gross lesions and visible tumors.

### 2.10 Calculation of the tumor spectrum

The tumor spectrum is used to reflect the distribution of different types of tumors. When calculating, all types of tumors that appear in the experiment should be counted according to the clear observation indicators and tumor definition criteria. Subsequently, the proportion of each type of tumor in the total occurrence of tumors can be calculated through the formula “Tumor Incidence Rate = (The number of specific tumors/The total number of tumors in this group of mice) × 100%”, to clearly present the distribution characteristics of tumors in each group of mice.

### 2.11 Statistical analysis

All statistical analyses were performed using GraphPad Prism 7.0., including mean calculations and correlation analyses. Results were presented as means ± standard deviations (SD). Comparative survival analyses were performed using the Log‐Rank test. *p* < 0.05 was considered as statistically significant.

## 3 RESULTS

### 3.1 The generation of NF‐hHRAS knock in mouse model

**FIGURE 1:**
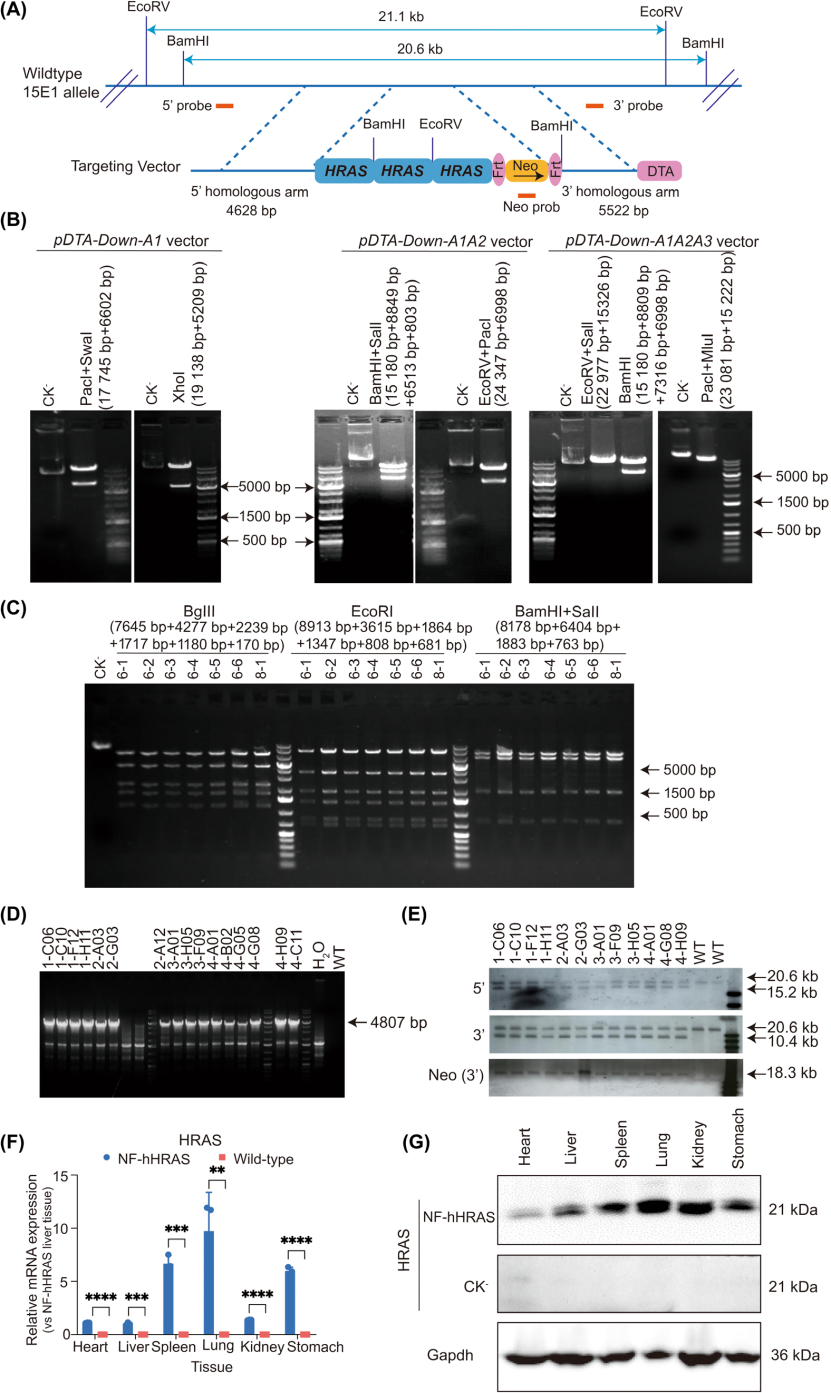
Establishment and verification of NF‐hHRAS mouse model. (A) The schematic diagram of the targeting vector of NF‐hHRAS knock‐in mouse model. (B) Restriction digestion analysis of pDTA‐down‐A1 vector, pDTA‐down‐A1A2 vector and pDTA‐down‐A1A2A3 vector. (C) Restriction digestion analysis of the correct pDTA‐down‐A1A2A3 vector with *BgIII*, *EcoRI*, and *BamHI + Sall* for further identification. (D) Long PCR identification of the Genomic DNA from positive ES cells clones. The expected PCR products were 4807 bp with primers PCR‐F1: GGCTTGACTGCCTGGGTGTTG, PCR‐R1 CTGCAATTGGTCACGTGGCTG; (E) Southern blot analysis of the Genomic DNA from ES cells. ES cell genomic DNA was digested with *BamHI*, the expected bands were 20.6 kb and 15.2 kb for 5′‐end probe, and the expected bands were 20.6 kb and 10.4 kb for 3′‐end probe. The transcription and translation of *hHRAS* gene mRNA in NF‐hHRAS mice detected by relative RT‐qPCR (F, *n* = 3) and Western blot (G, *n* = 2). The data were shown as the mean ± SEM for three independent experiments and were normalized to the corresponding *Gapdh* levels. ^ns^Not significant; **p < 0.01; ***p < 0.001; ****p < 0.0001.

The schematic diagram of the targeting vector of NF‐hHRAS knock in mouse was shown in Figure 1A. To achieve a higher expression level of the *HRAS* gene (Gene ID: 3265), we constructed a targeting vector containing three tandem copies of *HRAS* genes, with a total length of approximately 21 kb. To ensure site‐specific insertion at the mouse chromosome 15E1 locus, homologous arms of 4.6 kb and 5.5 kb were added to the 5′‐and 3′‐ends of the *HRAS* gene sequence, respectively. Additionally, *Neo* gene was incorporated at the 3′‐end of the construct to facilitate the identification and screening of positive stem cell clones.

We initially synthesized the full‐length genomic DNA of a single‐copy human *HRAS* gene and inserted it into the pDTA vector to generate the recombinant plasmid pDTA‐Down‐A1 (Figure 1B). Using in vivo BAC recombination, two additional copies of the *HRAS* gene, designated as fragments A2 and A3, were sequentially inserted downstream of the 3′‐end of A1, resulting in the construction of two new recombinant plasmid: pDTA‐Down‐A1A2 and pDTA‐Down‐A1A2A3. These vectors were digested by endonucleases and the expected bands were successfully obtained (Figure 1B). The correctly assembled vectors were selected for *HRAS* gene sequencing. Furthermore, the selected clones were digested with endonucleases *BgIII*, *EcoRI*, and *BamHI + Sall* for additional verification, and the expected fragments were clearly visualized (Figure 1C). Before electroporation, the karyotype of ES cells was examined, and it was confirmed that they were of the male type (XY) with a normal chromosome number (Table S1). 17 of ES cells clones, like 1‐C06 and 1‐C10, were validated by Long‐range PCR (Figure 1D) and subsequently verified by Southern blot (Figure 1E). The correct clones exhibited bands of 20.6 kb and 15.2 kb for the 5′‐end probe and bands of 20.6 kb and 10.4 kb for 3′‐end probes. The 1‐C06 clone were microinjected into blastocysts derived from BALB/c mice. Following implantation into pseudo‐pregnant C57BL/6J‐albino recipient mice, chimeric offspring were produced. Upon breeding with C57BL/6J female mice, 6 of 16 offspring, which carried the human *HRAS* genes but maintained a C57BL/6J genetic background, were identified through genotyping. Both RT‐qPCR and Western blot analyses demonstrated that the human *HRAS* were expressed in all tested tissues, with relatively higher expression levels observed in the spleen, lung and stomach (*p* < 0.05, Figure 1F,G). In summary, the *HRAS* gene was successfully inserted into the genome of C57BL/6J mice and exhibited non‐tissue‐specific expression. Thus, the KI.C57‐ras or NF‐hHRAS knock‐in mouse model were successfully established.

### 3.2 The genetic traits of NF‐hHRAS mice remain stable across generations

**FIGURE 2:**
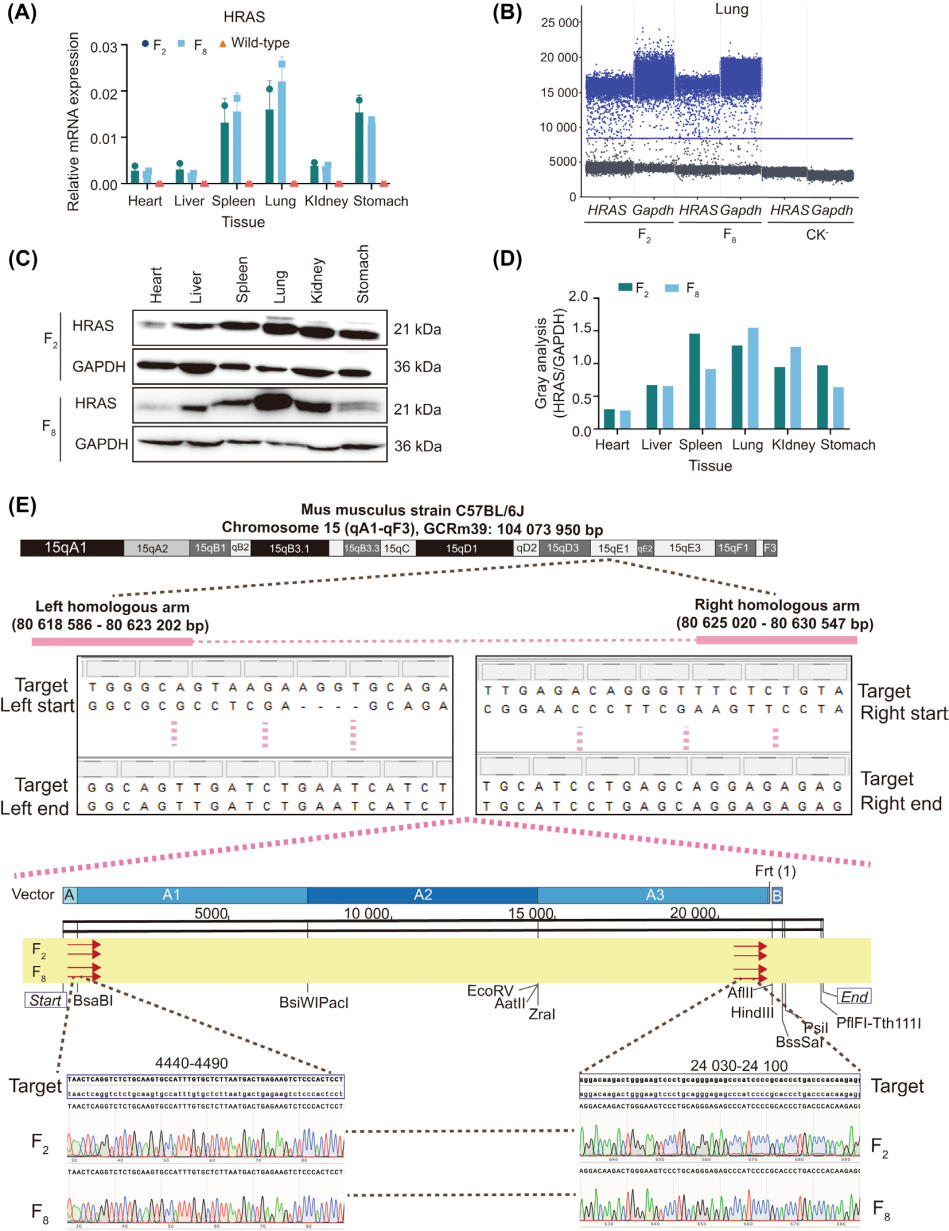
The genetic stability of NF‐hHRAS mice and integration site. (A) Comparison of the *HRAS* gene expression in the heart, liver, spleen, lung, kidney and stomach of F_2_ and F_8_ NF‐hHRAS mice (*n* = 3). Values were shown as the mean ± SD for three independent experiments. (B) Droplet Digital PCR analysis of the *HRAS* gene mRNA in the lung of F_2_ and F_8_ generations (*n* = 3). (C, D) Western blot and Protein gray scale analysis of the HRAS protein in the heart, liver, spleen, lung, kidney and stomach of NF‐hHRAS mice (*n* = 3). (E) Identification of integration site of *HRAS* gene in NF‐hHRAS mice.

To confirm whether the transgene could be stably expressed across different generations of NF‐hHRAS mice, total RNA was isolated from the heart, liver, spleen, lung, kidney and stomach of both F_2_ and F_8_ generations of mice and analyzed by RT‐qPCR. It was observed that the expression levels of *HRAS* gene were consistent across all tested organs in both generations, but were significantly higher than those in wild‐type mice (Figure 2A). For more precise comparison, the *HRAS* gene expression level in the lungs were quantified using digital PCR. The results demonstrated that there were no significant differences in the expression levels of ether the *HRAS* gene or the reference *Gapdh* gene between the two generations (Figure 2B). Additionally, the translation of the HRAS protein was found to be similar between the two generations as well (Figure 2C,D).

To identify the integration site of the *HRAS* gene, the 5′‐end left (4628 bp) and 3′‐end right (5522 bp) homologous arms were aligned against the NCBI GeneBank database. The results revealed that these sequences mapped to chromosome 15 qA1‐qF3 region (GCRm39), with the left arm spanning from 80 618 586 bp to 80 623 202 bp, and the right arm spanning from 80 625 020 bp to 80 630 547 bp (Figure 2E). Then, DNA fragments at the junction sites of the 5′‐end and 3′‐end of the NF‐hRAS mouse genome, including samples from both F_2_ and F_8_ generations, were sequenced. The results showed that the DNA sequences of the two fragments (4440–4490 bp and 24 030–24 100 bp, Figure 2E) were entirely consistent with the mouse genomic DNA sequence of chromosome 15 (15E1 site), confirming that the human *HRAS* gene had been successfully inserted into the 15E1 locus. Notably, the sequencing results for the F_8_ generation were identical to those of the F_2_ generation, with no detectable differences observed (Figure 2E).

### 3.3 NF‐hHRAS mouse was susceptible to MNU carcinogen

The human *HRAS* gene is a classical proto‐oncogene, high expression in tissues may increase the risk of carcinogenesis in both humans and genetically modified mice, rendering animals more sensitivity to carcinogens.^22^, ^23^ It has been reported that rasH2 transgenic mice were highly susceptible to various carcinogens.^24^, ^25^ To minimize the impact of spontaneous tumors on the experimental results, F_1_ hybrid mice derived from C57BL/6J and BALB/c strains are commonly used. N‐Nitroso‐N‐methylurea (MNU) is frequently selected as a positive carcinogen for testing the carcinogenic potential of mouse models.^26^ To verify whether NF‐hHRAS mice were susceptible to carcinogens, MNU was at administered intraperitoneally at a dose of 75 mg/kg body weight per mouse, followed by a 26‐week observation period. The body weight, survival rate, surface mass, and tumor incidence were recorded and analyzed. The experimental design adhered to the guidelines outlined Organization for Economic Co‐operation and Development (OECD) 451 and ICH S1B.

### 3.4 NF‐CB6F1(hHRAS ^+/−^) mice showed lower survival rate and weight gains

**FIGURE 3:**
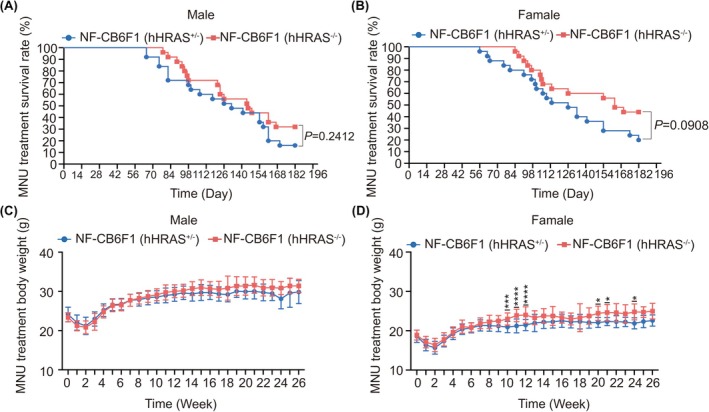
Body weight and survival rate of mice treated by MNU. (A, B) Survival rate of mice treated by MNU. The survival rate of male NF‐CB6F1(hHRAS^+/−^) mice and NF‐CB6F1(hHRAS^−/−^) was 16% and 32% at 26 weeks after dosing, the survival rate of female NF‐CB6F1(hHRAS^+/−^) mice and NF‐CB6F1(hHRAS^−/−^) was 16% and 44%. (C, D) Body weight of mice treated by MNU. *p < 0.05; ***p < 0.001; *****p < 0.0001.

Hybrid F_1_ mice exhibited two genotypes, NF‐CB6F1(hHRAS^+/−^) and NF‐CB6F1(hHRAS^−/−^). As shown in Figure 3A,B, both male (*p* = 0.2412) and female (*p* = 0.0908) NF‐CB6F1(hHRAS^+/−^) showed lower survival rate. By the 26‐week endpoint, only 4 male (16%) and 5 female (20%) remained alive, with the earliest deaths occurring on day 65 for males and day 56 for females. In contrast, for NF‐ CB6F1(hHRAS^−/−^) mice, 8 male (32%) and 11 female (44%) survived, with the earliest deaths observed on day 78 for males and day 84 for females, respectively. The onset of the first deaths was significantly delayed in NF‐CB6F1(hHRAS^−/−^) mice compared to NF‐CB6F1(hHRAS^+/−^) mice.

As shown in Figure 3C,D, MNU treatment significantly suppressed weight gain in both male and female NF‐CB6F1(hHRAS^+/−^) mice (*p* < 0.05). Compared to wild‐type NF‐CB6F1(hHRAS^−/−^) mice, NF‐CB6F1(hHRAS^+/−^) mice showed increased weight loss from weeks 8–26. Starting from the 10th week, a visible difference in body weight was observed (*p* ≤ 0.01). These results indicated that NF‐CB6F1(hHRAS^+/−^) mice were highly susceptible to the carcinogen MNU, as evidenced by lower survival rates, more pronounced weight loss, and earlier mortality.

### 3.5 NF‐CB6F1(hHRAS ^+/−^) mice showed more macroscopic lesions

**FIGURE 4:**
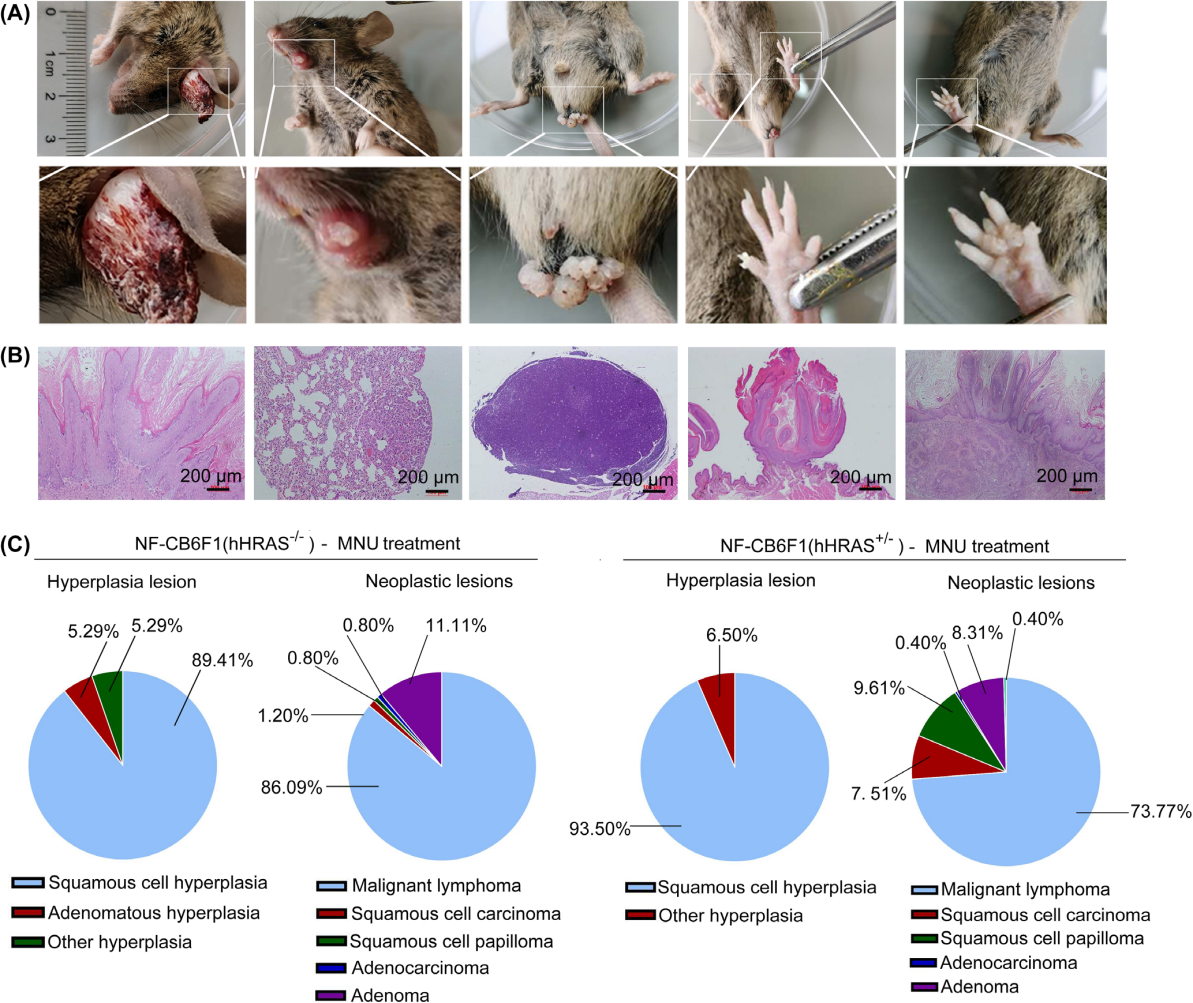
The macroscopic lesions, histopathological changes and tumor spectrum in NF‐CB6F1(hHRAS^+/−^) mice treated with MNU. (A) Skin masses and nodules near to the ears, perioral region, tongue, genital skin, and paws of NF‐CB6F1(hHRAS^+/−^) mice. (B) Histopathological features of MNU induced tumors in NF‐CB6F1(hHRAS^+/−^) mice, from left to right, they were squamous cell papilloma of the stomach, adenoma of the lung, lymphoma of the lymph node, squamous cell papilloma of the pudendum skin, and squamous cell carcinoma of the stomach (×200, scale bar: 200 μm). (C) The proportion of neoplastic lesions and hyperplasia lesions in the NF‐CB6F1(hHRAS^+/−^) mice and NF‐CB6F1(hHRAS^−/−^) mice.

The types, locations and frequency of macroscopic lesions were summarized in Table 2. Following MNU administration, gross lesions were observed to increase in size of multiple organs, including the thymus, spleen, intestines, lymph nodes, kidney, liver and salivary gland. Additionally, masses or nodules developed in various organs, such as the forestomach, perineal region, neck, back and paw. Diverse neoplasms were also detected on the skin near the nose, tongue, eyes, ears and oral cavity (Figure 4A). Other gross lesions associated with MNU administration were also observed, such as thymus atrophy and pleural effusion, in NF‐CB6F1(hHRAS^+/−^) mice. Similar macroscopic lesions could also be detected in MNU‐treated NF‐CB6F1(hHRAS^−/−^) mice; however, the frequency was either lower or absent (Table 2). It is noteworthy that no macroscopic lesions were found in the organs of NF‐CB6F1(hHRAS^+/−^) mice in the solvent control group. MNU‐treated NF‐CB6F1(hHRAS^+/−^) mice developed genotype‐specific lesions, including masses in the forestomach, perineal region, skin, and claws, as well as salivary gland enlargement. Both male and female NF‐CB6F1(hHRAS^+/−^) mice demonstrated susceptibility to the carcinogen, although males exhibited a slightly higher degree of susceptibility.

**TABLE 2:** Macroscopic findings of MNU treated mice.

Dose		MNU 75 mg/kg			
Animals		CB6F1 (hHRAS^+/−^)	CB6F1 (hHRAS^+/−^)	CB6F1 (hHRAS^−/−^)	CB6F1 (hHRAS^−/−^)
Sex		Male	Female	Male	Female
Numbers of animals		25	25	25	25
Organs	Symptoms	Numbers of animals affected			
Forestomach	Nodules	23	20	1	1
	Mass	2	1	0	0
Thymus	Enlarged	11	12	15	12
	Atrophy	5	2	2	
Perineal skin	Nodules	21	5	0	0
Skin (nose, oral, tongue, eye, ear)	Nodules	10	11	0	0
Claw	Nodules	12	3	0	0
Spleen	Enlarged	11	9	5	8
Lymph node	Enlarged	9	7	4	1
Intestines	Distention	8	6	4	1
Liver	Enlarged	8	6	5	3
Kidney	Enlarged	6	0	2	0
Pleural	Effusion	5	2	0	1
Lung	White color	3	7	4	1
Neck	Mass	1	1	0	1
Back	Mass	0	1	2	0

### 3.6 NF‐CB6F1(hHRAS ^+/−^) mice showed a higher rate of microscopic lesions

All animals subjected to emergency and terminal necropsy at the end of 26 weeks were collected for histopathological examination (Figure 4B). The tumor incidence in MNU‐treated NF‐CB6F1(hHRAS^+/−^) mice reached 100% in both females and males (25/25 each), indicating that all CB6F1(hHRAS^+/−^) mice developed tumors. This rate was higher than that observed in NF‐CB6F1(hHRAS^−/−^) mice, which was 80% (20/25) in females and 84% (21/25) in males (Table 3). Malignant lymphoma was the predominant neoplastic lesion, detected in multiple organs and tissues across most animals (Tables 3 and 4). Squamous cell carcinoma, squamous cell papilloma and squamous cell hyperplasia were observed in forestomach. Squamous cell papilloma and squamous cell hyperplasia were also observed in skin, pudendum skin, and tongue. Adenocarcinoma was identified in the intestine, whereas adenoma was detected in both the lungs (bronchiolo‐alveolar) and the intestines (Table 4).

**TABLE 3:** Microscopic findings of MNU treated mice.

Dose	MNU75mg/kg			
Animals	CB6F1 (hHRAS^+/−^)	CB6F1 (hHRAS^+/−^)	CB6F1 (hHRAS^−/−^)	CB6F1 (hHRAS^−/−^)
Sex	Male	Female	Male	Female
No. animals	25	25	25	25
Microscopic findings	Number of animals affected			
Malignant lymphoma	14	18	16	16
Squamous cell carcinoma	14	7	0	2
Squamous cell papilloma	16	5	0	2
Squamous cell hyperplasia	9	14	8	10
Adenocarcinoma	2	0	2	2
Adenoma	9	10	10	3

**TABLE 4:** Microscopic findings of MNU treated mice and primary organs.

Dose		MNU75mg/kg			
Animals		CB6F1 (hHRAS^+/−^)	CB6F1 (hHRAS^+/−^)	CB6F1 (hHRAS^−/−^)	CB6F1 (hHRAS^−/−^)
Sex		Male	Female	Male	Female
No. animals		25	25	25	25
Organs	Microscopic findings	Number of animals affected			
Heart	Malignant lymphoma	10	6	11	12
Liver	Malignant lymphoma	9	7	8	9
Spleen	Malignant lymphoma	9	4	7	10
Lung	Malignant lymphoma	12	9	9	13
Kindey	Malignant lymphoma	10	6	9	6
Thymus	Malignant lymphoma	5	11	9	4
Bone marrow	Malignant lymphoma	11	6	7	8
Intestine	Malignant lymphoma	0	1	1	2
Lymph node	Malignant lymphoma	5	5	6	2
Reproductive system	Malignant lymphoma	2	5	5	6
Nose	Malignant lymphoma	4	4	4	2
Trachea	Malignant lymphoma	2	0	1	0
Esophagus	Malignant lymphoma	3	1	2	1
Pancreas	Malignant lymphoma	2	1	0	1
Gallbladder	Malignant lymphoma	0	3	3	1
Forestomach	Squamous cell carcinoma	14	7	0	2
Forestomach	Squamous cell papilloma	7	3	0	2
Skin	Squamous cell papilloma	3	1	0	0
Pudendum Skin	Squamous cell papilloma	9	0	0	0
Tongue	Squamous cell papilloma	1	1	0	0
Forestomach	Squamous cell hyperplasia	7	14	8	10
Skin	Squamous cell hyperplasia	0	1	1	0
Pudendum skin	Squamous cell hyperplasia	2	1	0	0
Intestine	Adenocarcinoma	3	0	2	2
Lung	Adenoma	8	10	8	3
Intestine	Adenoma	1	0	0	0

Table 4 demonstrated that various tumors were detected in multiple organs of NF‐CB6F1(hHRAS^+/−^) mice, highlighting the susceptibility of this mouse model. In NF‐CB6F1(hHRAS^−/−^) mice, the incidence of tumors in forestomach, skin, pudendum and lungs were lower or absent, indicating reduced susceptibility. To enable a more precise comparison, we calculated the incidence rates of each tumor type in the animals. The incidence of malignant lymphoma was 73.77% in NF‐CB6F1(hHRAS^+/−^) and 86.09% in NF‐CB6F1(hHRAS^−/−^) mice, respectively (Figure 4C). However, the incidences of squamous cell carcinoma and squamous cell papilloma were significantly higher in NF‐CB6F1(hHRAS^+/−^) mice compared to NF‐CB6F1(hHRAS^−/−^) mice (7.51% vs 1.2%, 9.61% vs 0.80% respectively). The incidence of MNU‐induced proliferative lesions, including squamous cell carcinoma, squamous cell papilloma, and squamous cell hyperplasia, was calculated for both mouse strains (Figure 4C). The proportion of squamous cell hyperplasia in NF‐CB6F1(hHRAS^+/−^) mice was 93.50%, while other proliferative lesions constituted 6.5%. In NF‐CB6F1(hHRAS^−/−^) mice, the proportion of squamous cell hyperplasia, adenomatous hyperplasia and other types of hyperplasia were 86.09%, 5.29% and 5.29% respectively. Moreover, the incidence of squamous cell hyperplasia in the forestomach of NF‐CB6F1(hHRAS^+/−^) mice was significantly higher than that in hHRAS^−/−^ mice. In summary, the microscopic findings indicate that NF‐CB6F1(hHRAS^+/−^) mice are more susceptible to developing such lesions compared to their littermates, NF‐CB6F1(hHRAS^−/−^) mice.

### 3.7 The tumor incidence of NF‐CB6F1(hHRAS ^+/−^) mice

**FIGURE 5:**
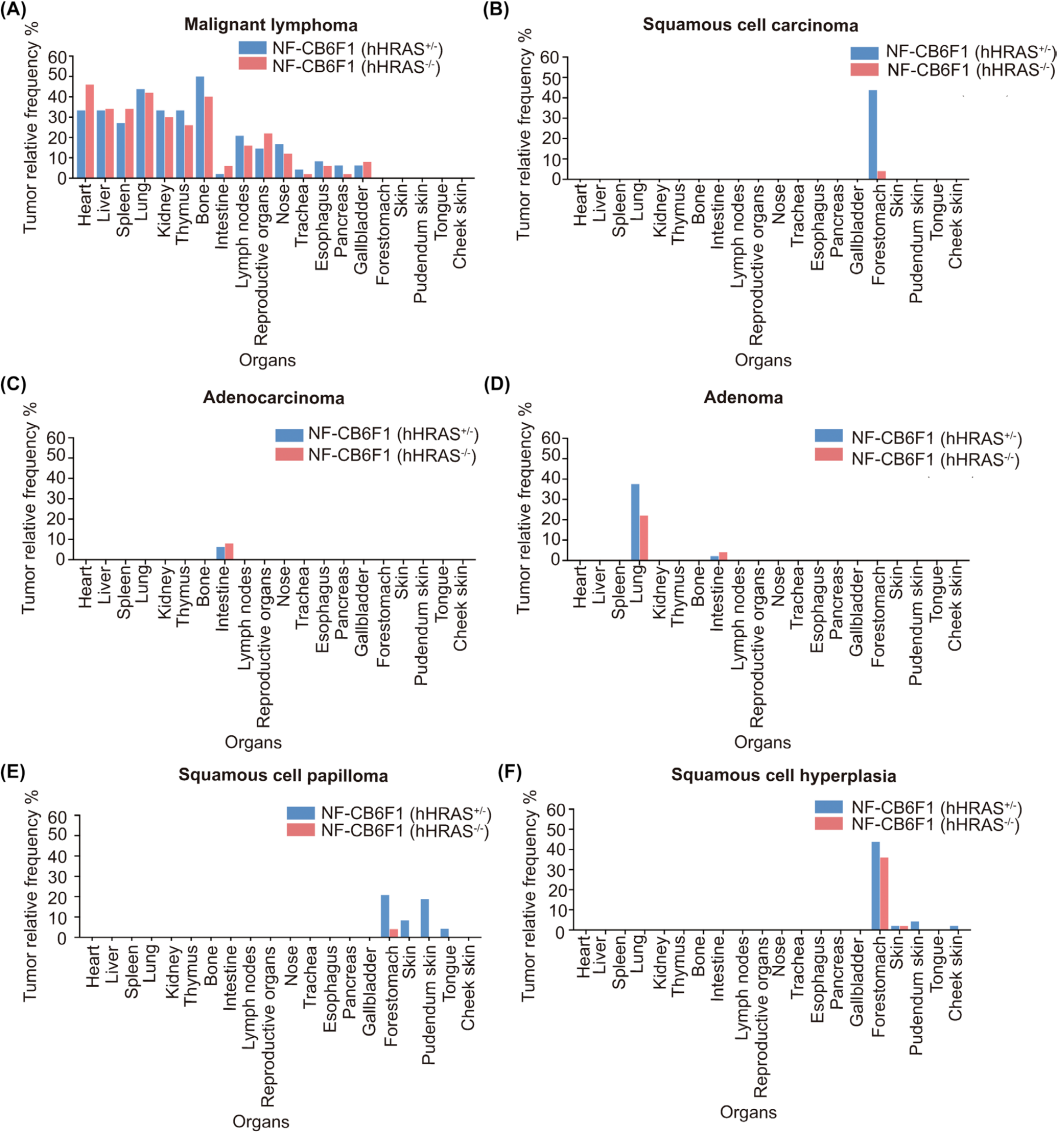
Tumor spectrum of NF‐CB6F1(hHRAS^+/−^) and NF‐CB6F1(hHRAS^−/−^) mice. (A–F) The incidence of different tumors in various tissues and organs of NF‐CB6F1(hHRAS^+/−^) and NF‐CB6F1(hHRAS^−/−^) mice treated with MNU.

To visually present the types and occurrence rates of tumors across various organs, we calculated the incidence of each tumor in different organs (Figure 5A–F). Malignant lymphoma was observed in multiple organs in both NF‐CB6F1(hHRAS^+/−^) and NF‐CB6F1(hHRAS^−/−^) mice, including the heart, liver, spleen, thymus, kidney, bone, lymph nodes, reproductive organs, nose, trachea, esophagus, pancreas and gallbladder (Table 4). The incidence rates of malignant tumors in NF‐CB6F1(hHRAS^+/−^) and NF‐CB6F1(hHRAS^−/−^) mice were comparable (Figure 5A). Malignant lymphoma is likely to originate in the spleen, thymus, bone and lymph nodes, whereas lymphoma observed in other tissues or organs may represent metastic spread from primary sites.^27^, ^28^ Squamous cell carcinoma was observed exclusively found in the forestomach, with a higher incidence in hHRAS‐positive mice (Figure 5B), and represented one of the characteristic tumor types in the NF‐hHRAS carcinogenic mouse model. Squamous cell papilloma predominantly occurred in the forestomach, skin, and pudendum skin of hHRAS‐positive mice (Figure 5E), whereas adenoma was primarily detected in the lungs, with a relatively high incidence (Figure 5D). The histopathological findings in the forestomach were corresponding to the incidence of the gross lesions of the two mice.

### 3.8 NF‐hHRAS mouse showed low spontaneous tumors rate

The spontaneous tumor incidence is a critical parameter for assessing the reliability of carcinogenic mouse models, as a high rate of spontaneous tumors may confound experimental results. In this study, we monitored 50 KI.C57‐ras mouse models (25 males and 25 females, treated with solvent only) over a 6‐month period. At week 21, one female mouse developed vulvar nodules accompanied by dyspnea. Histopathological examination confirmed the presence of both forestomach squamous cell papilloma and splenic lymphoma. However, no tumor lesions were observed in the remaining 49 mice. Therefore, the spontaneous tumor incidence was 2% (1/50). This tumor incidence rate should be updated as more background data is available.

## 4 DISCUSSION

Genome editing technology was initially developed in the late 1980s. Over the past decade, it has undergone rapid advancement. The precise knock‐in of large DNA fragments remains one of the major obstacles that scientists aim to address. In this study, we successfully established a novel carcinogenic mouse model, KI.C57‐ras (alternatively named NF‐hHRAS), utilizing embryonic stem cell targeting technology to insert a 21 kb large fragment of human *HRAS* genomic DNA (Figure 1) into the C57BL/6J mouse genome. NF‐hHRAS mice have stable genetics and high expression levels of the *HRAS* transgene (Figure 2). It has been propagated to the eighth generation, with consistent phenotypes observed across all generations of the model kept consistent. Moreover, the long‐read whole‐genome sequencing is currently being conducted on the NF‐hHRAS models to further confirm that no mutations have occurred in the *HRAS* gene.

Through target vector homologous arm sequencing and alignment comparison using the NCBI gene bank, we revealed that the *HRAS* transgene was inserted into the mouse chromosome 15E1 site, specifically positioned between 80 623 202 bp and 80 625 020 bp (Figure 2E). Previously, the FISH technique was frequently employed to identify the foreign insertion site; however, this method lacked precision and could only localize foreign genes to a lager region.^22^ Next, by the whole‐genome sequencing of NF‐hHRAS mice, we aim to precisely map the insertion site of the *HRAS* gene at the base‐pair level. In a word, we have innovatively accomplished the site‐specific insertion of a large DNA fragment.

The insertion site of *HRAS* transgene is of particular concern because it directly influences the expression level, genetic stability of the mouse models, and the transcription and translation of host genes in mice, ultimately affecting the phenotype of the genetically modified mouse. The exploration of the *Rosa26*, *Hip11* as safe harbor sites aims to avoid the adverse effects of insertion sites on transgenes expression and function.^29^ In transgenic mouse model,^30^, ^31^, ^32^ foreign genes are randomly integrated into the genome, often resulting in altered expression profile of the host genes and causing instability in genetic inheritance or even failure in breeding.

In addition, the selection of the promoter, gene copy number, and gene tandem arrangement also influence transgene expression, thereby affecting the phenotype of genetically modified mouse models.^33^, ^34^ To achieve a high and intrinsic expression profile, we innovatively linked three tandem copies of the *HRAS* gene while retaining its native promoter. Moreover, the sequence of *HRAS* gene was intact, unlike the transgene in rasH2 mice, which contains a mutation.^35^ According to our knowledge, no similar targeting strategy has been reported, which is attributable to our highly efficient ES cell targeting system.

We further revealed that NF‐hHRAS mice presented significantly increased susceptible to carcinogens. Upon exposure to the MNU carcinogen, the initial death time of NF‐hHRAS mice was earlier than that of wild‐type littermates (Figure 3, day 65 vs. day 78 for male and day 56 vs. day 84 for female). Additionally, 100% of NF‐hHRAS occurred tumors, whereas only approximately 80% of wild‐type littermates occurred (Table 3). Moreover, approximately 84% of male NF‐hHRAS mice developed specific tumors, such as squamous cell carcinoma or squamous cell papilloma, whereas no wild‐type mouse exhibited (Table 4). The tumor types (Table 3) and target organs were highly diverse (Figures 4 and 5), underscoring the high sensitivity to the carcinogen while maintaining a low spontaneous tumor incidence rate (1/50). Notably, key parameters such as tumor type and incidence in NF‐hHRAS are consistent with those of the recognized rasH2 mice. Furthermore, the characteristic tumor of NF‐hHRAS model was forestomach squamous cell carcinoma, as opposed to malignant lymphoma, a finding that is consistent across both sexes and is exclusively observed in transgenic hHRAS‐positive mice. Consistent with the rasH2 models,^36^ the gender‐related disparity in tumor incidence observed in this study is anticipated to gradually diminish as the number of experimental replicates increases.

Carcinogenicity assessment represents one of the critical components of non‐clinical safety evaluation for drugs and medical devices. Since the introduction of the ICH S1B in 1997, the carcinogenicity assessment method utilizing genetically modified mouse models has been extensively adopted worldwide.^37^, ^38^ The rasH2 transgenic mouse model, which harbors the recombinant human *c‐Ha‐ras* gene and was established by Japanese scientists, is extensively utilized in global non‐clinical safety evaluations of drugs and has garnered broad recognition from regulatory bodies such as U.S. Food and Drug Administration (FDA), Organization for Economic Co‐operation and Development (OECD), World Health Organization (WHO), and ICH. The evaluation results are widely used to support applications for new drug approvals. However, the recombinant transgene of rasH2 mouse model partially originates from melanoma patients and contains mutations.^39^ This model was established in 1990 used traditional transgenic technology, which involves random insertion sites and unclear copy numbers of the transgene. Although the insertion sites and transgene copy numbers were subsequently verified, the results remain questionable.^22^, ^35^ Moreover, this mouse model is relatively thin and has a short tumor‐bearing survival time. Following administration of 1000 mg/kg urethane, all animals succumbed within less than 6 months, which is the endpoint required by the ICH S1B guideline. The earlier mortality may not be conducive to fully unveiling the potential carcinogenic risks of the test substance. Consequently, it is imperative to establish novel carcinogenic mouse models for non‐clinical carcinogenicity assessments of drugs.

In conclusion, this study successfully achieved the precise insertion of large DNA fragments via stem cell targeting technology. The novel genetically modified NF‐HRAS mouse has clearly defined transgene insertion sites and copy numbers. This mouse model demonstrates excellent genetic stability, a stable phenotype, high susceptible to carcinogens, and a low incidence of spontaneous tumors, indicating its potential for wide use in non‐clinical safety evaluation of drugs.

## AUTHOR CONTRIBUTIONS

**Susu Liu:** Data curation; formal analysis; methodology; resources; writing – original draft. **Yanwei Yang:** Formal analysis; methodology. **Guitao Huo:** Formal analysis; methodology. **Hao Yang Zhao:** Methodology; resources. **Chen Ling:** Data curation; formal analysis. **YuYa Wang:** Data curation; formal analysis; methodology; writing – original draft. **Shijie Zhai:** Methodology; resources. **Xiaowei Sun:** Methodology; resources. **Wenda Gu:** Methodology; resources. **Yuan Cao:** Methodology; resources. **Wei Gong:** Supervision. **Sanlong Wang:** Conceptualization; writing – review and editing. **Changfa Fan:** Conceptualization; writing – original draft; writing – review and editing.

## FUNDING INFORMATION

This work was jointly supported by the National Key R&D Program of China (2023YFC3402000) and National Institutes for Food and Drug Control, State Key Laboratory of Drug Regulatory Science (2023SKLDRS0124).

## CONFLICT OF INTEREST STATEMENT

The authors declare that they have no competing interests.

## ETHICS STATEMENT

Wild‐type C57BL/6J mice and genetically modified mice were supplied by the Institute for Laboratory Animal Resources, National Institute for Food and Drug Control (NIFDC, Beijing China). All animal experiments were approved by the NIFDC Institutional Animal Care and Use Committee [approval number: NIFDC2021(B)003]. The license number of the Animal Use Certificate issued by the Science & Technology Department of China (Beijing, China) was SYXK 2022‐0002. All animal experiments strictly adhered to internationally accepted principles for laboratory animal care and use, following both the ARRIVE and American Veterinary Medical Association (AVMA) guidelines.

## Supporting information


**Data S1:** Supporting information.
