# Supplementary material for: A novel carcinogenic mouse model by site‐directed insertion of tandem human HRAS large DNA fragment into 15E1 site
Source: Animal Model Exp Med. 2025 Nov 12;8(11):1983–96. doi: 10.1002/ame2.70086 (PMC12746206; doi:10.1002/ame2.70086)
Supplement: Supplementary file 1 — Data S1: Supporting information. [file AME2-8-1983-s001.docx]

**Supplementary Tables**

**Supplementary Table 1 Karyotype analysis report-G-banding**

| Clones | The actual number of counts | | | | Rate of  abnormal  karyotype |
| --- | --- | --- | --- | --- | --- |
|  | 2n<40 | 2n=40 | 2n>40 | Abnormalities in chromosome  structure |  |
| 1-C06 2# | 3 | 26 | 1 | 0 | 13.4 % |
| 2-A03 2# | 3 | 4 | 18 | 0 | 84.0 % |
| 1-F12 2# | 2 | 27 | 1 | 0 | 10.0 % |
| 1-C10 2# | 2 | 30 | 0 | 0 | 6.2 % |
| 4-A01 2# | 2 | 28 | 0 | 0 | 6.7 % |
| 3-A01 2# | 2 | 27 | 1 | 2 | 10.0 % |
